# Comparative Analysis of the Mechanical Behaviour of PEF and PET Uniaxial Stretching Based on the Time/Temperature Superposition Principle

**DOI:** 10.3390/polym13193295

**Published:** 2021-09-27

**Authors:** Emilie Forestier, Christelle Combeaud, Nathanael Guigo, Guillaume Corvec, Christophe Pradille, Nicolas Sbirrazzuoli, Noelle Billon

**Affiliations:** 1 MINES Paris Tech, PSL Research University, CNRS, Centre de Mise en Forme des Matériaux (CEMEF), UMR 7635, CEDEX, 06904 Sophia Antipolis, France; emilie.forestier@mines-paristech.fr (E.F.); guillaume.corvec@mines-paristech.fr (G.C.); noelle.billon@mines-paristech.fr (N.B.); 2 Institut de Chimie de Nice (ICN), Université Cote d’Azur, CNRS, UMR7272, CEDEX 2, 06108 Nice, France; nathanael.guigo@univ-cotedazur.fr; 3 Mat Xper, 19 Traverse du Barri, 06560 Valbonne, France; christophe.pradille@mat-xper.com

**Keywords:** biobased thermoplastics, poly(ethylene 2,5-furandicarboxylate) (PEF), poly(ethylene terephthalate) (PET), time/temperature principle, strain-induced crystallization, uniaxial stretching

## Abstract

Poly(ethylene 2,5-furandicarboxylate), PEF and poly(ethylene terephthalate), PET, are two polyesters with close chemical structures. It leads to similar thermal, mechanical and barrier properties. In order to optimize their stretching, a strategy based on the time/temperature principle is used. The building of master curves, in the linear visco-elastic domain, allows the identification of the experimental conditions for which the two materials are in the same physical state. The initial physical state of the materials is important as, to fit with the industrial constrains, the polymers must reach high level of deformation, and develop strain induced crystallization (SIC). In this paper, the screening of the forming range is described, as well as the mechanical response depending on the stretching settings. Moreover, the same mechanical response can exist for PEF and PET if the same gap from the α-relaxation exists.

## 1. Introduction

Poly(ethylene 2,5-furandicarboxylate), PEF and poly(ethylene terephthalate), PET, are two polyesters of rather equivalent mechanical, thermal and barrier properties [1,2,3,4,5,6,7,8,9]. PEF is considered as the bio-based counterpart of PET that could be used in industrial applications such as stretching, spinning or blowing. Despite these similitudes, they behave slightly differently because of their chain architecture which makes their comparison of high scientific interest. Indeed, the main structural difference between PET and PEF is the presence of the benzene ring in the PET constitutive unit, while it is a furan ring for PEF. It induces different local mobility: contrary to the benzene ring, the activation of the flipping motion of the furan ring is more difficult [1]. Nevertheless, when they are stretched from the amorphous state, both materials can exhibit strain induced crystallization and a drastic mechanical strain hardening [10,11,12]. It can be achieved when the materials are stretched in a rubbery-like state, where stretch ability is maximum. In industrial processes such as films stretching, injection stretch blow molding (ISBM) or thermoforming, polymers must undergo draw ratio of 4, or more, in two directions (biaxial extensions of 8 to 12). Strain hardening and the ability to develop strain induced crystallization (SIC) are key issues that must be controlled in such processes. Therefore, the ability of these two polyesters for being stretched to high levels, and their ability to develop SIC need a comparison to better understand the general behaviour of aromatic polyesters.

This phenomenon was widely explored for PET [13,14,15,16,17,18,19,20,21,22,23,24,25,26] since the 70’s. There are much less investigations concerning PEF whose technical development is very recent [10,11,12,27,28,29,30]. In PET, SIC is progressively developed [19,21,22,25,31]. It is supposed that, before the crystal apparition, there is the existence of an intermediate phase, named mesophase. This oriented and organized phase acts as a crystal precursor [19,21,32,33,34,35,36,37,38]. The stable crystal, with all its symmetry and periodicities, needs a relaxation step to appear, and is formed after the stretching end. The mesophase is formed from the stretching beginning [32,34,35,39]. Thus, the obtained microstructure is directly dependent on the stretching settings and on the post-stretching treatment [40]. In PEF, the presence of a mesophase prior to crystallization is postulated [27], but according to our recent works [10,11,12], we have suggested that PEF microstructural development is more binary: crystal may or may not exist. The crystalline structures are reported to be triclinic for PET [27] and monoclinic for PEF [28,29]. In conditions leading to SIC, the macroscopic behaviour of the polymers is sensitive to the temperature and the strain rate, in a very significant and combined manner (see for example [41]). Additionally, as polymers are not crosslinked, their behaviour results from the combination of hyper-elasticity (as expected in that range) and of viscoelasticity. Consequently, the stretching range has to be defined in terms of temperature/strain rate sets of conditions (*T, έ*). It was already reported that close to *T_α_* the combined dependencies of temperature and strain rate could be considered via an unified manner, based on the extension of classical time/temperature superposition principle for different materials (PMMA, PA66, PET and PEF) [10,11,12,30,40,41,42,43,44]. However, due to differences in the *α*-transition temperature between PEF and PET, the precise processing range must be adjusted to account for the intrinsic chain dynamic. This paper aims at assessing the stretch abilities of amorphous PEF and PET, by extending and validating the “time/temperature” superposition principle identified in the low deformation domain. DMTA analysis allows the building of elastic modulus master curve which could be representative of the polymer behaviour in the large deformation domain. The strategy used is well described in [41]. In a first time, the mechanical behaviour of each material has been investigated by DMTA, where master curves for a reference temperature have been built up. In a second time, cold crystallization in quiescent conditions has been investigated to be certain that, over the stretching duration, the polymer remains totally amorphous. DSC analyses were performed in that way. Then, to assess the stretch abilities of amorphous PET and PEF six different sets of strain rate and temperature were used to perform uniaxial tension up to high deformations and until rupture. Those sets were chosen to address three different equivalent strain rates at a reference temperature, not far from *T_α_*. The objective is to scan conditions within the entire rubbery-like zone observed on the elastic modulus master curve associated to each polymer. By doubling the technological sets of each equivalent strain rate at the reference temperature, one expects to validate the time/temperature superposition principle at high strains. By using three different equivalent strain rates, corresponding to three different positions on the master curve, one expected to scan most of the processing range that could be found in industrial processes. Digital Image Correlation (DIC) was used to address true tensile stress and strain. The stretching protocol proposed is defined to observe the strain hardening apparition (its onset is defined by the natural draw ratio, NDR), and possibly to allow the materials to develop an organized and oriented microstructure.

Finally, after the stretching, the samples are air-quenched and to complete this study, an overview of the induced microstructures is presented. 

## 2. Materials and Methods

### 2.1. Materials

Poly(ethylene 2,5-furandicarboxylate) was synthetized from the direct esterification and melt- solid state polycondensation (SSP) of monoethylene glycol and 2,5-furandicarboxylic acid (FDCA) produced by Avantium Renewable Polymers (Amsterdam, the Netherlands). Extruded PEF sheets with a thickness of 700 μm have been provided. Samples were extracted in the extrusion direction to minimize thickness variation. A commercially available PET grade (RamaPET N180^®^ from Indorama, Bangkok, Thailand), supplied by Sidel company (Le Havre, France), was extruded into PET sheets by Avantium Renewable Polymers (Amsterdam, Netherlands) with a thickness of 700 μm. Samples were extracted in the extrusion direction too. 

Extrusions were performed according to state of the art after drying to avoid hydrolysis and degradation. Samples were stored under vacuum, in an aluminium coated bag, in the freezer (−18 °C) to avoid water absorption and physical aging. Consequently, materials were tested dry, as processed, without any pre-conditioning.

### 2.2. DMTA Measurements

All DMTA experiments were conducted in tension using a Mettler-Toledo^®^ DMA 1, Greifensee, Switzerland. The sample dimensions were 5 × 4 × 0.7 mm^3^ for amorphous samples, and around 5 × 3 × 0.3 mm^3^ for stretched samples. Before each test, the sample underwent a preload of 1 N. Temperature scans were performed between −150 °C and 200 °C, at a heating rate of 1 °C/min, with a displacement amplitude of 5 μm (i.e., strain of 0.1%), in Auto-Tension mode. Temperature scans were carried out at a frequency of 1 Hz. Three measurements have been performed for each mechanical tests, the curves were well superimposed.

### 2.3. DSC Measurements

DSC measurements are performed on a Mettler Toledo^®^ DSC 1 (Greifensee, Switzerland) equipped with the STAR^®^ software. Aluminium pans of 40 µL are used. The samples weight is of approximatively 3 mg.

From their glassy state, the amorphous samples have been submitted to isothermal programs of various temperatures (between 90 °C and 180 °C) and durations (between 1800 s and 12,000 s). Then, they have been rapidly quenched (50 °C/min) and heated at 20 °C/min from 25 °C 250 °C. To measure crystal ratios, the stretched samples have been heated from 25 °C to 300 °C at 10 °C/min. Equation (1) has been used to calculate the crystal ratio obtained after the melting.

(1) $$\chi =\frac{\Delta H_{m}}{\Delta H_{m}^{0}}\;\;$$

with Δ*H_m_* the melting enthalpy and Δ*H_m_*^0^ the equilibrium melting enthalpy, taken at 140 J × g^−1^ for PEF and PET [11,30,45].

### 2.4. Stretching Conditions Determination

#### 2.4.1. Determination of the Forming Range

To perform efficient stretching on PEF and PET, an original protocol has been established, and previously published [10,11,12]. More details concerning this protocol are given in the present article.

**Figure 1:**
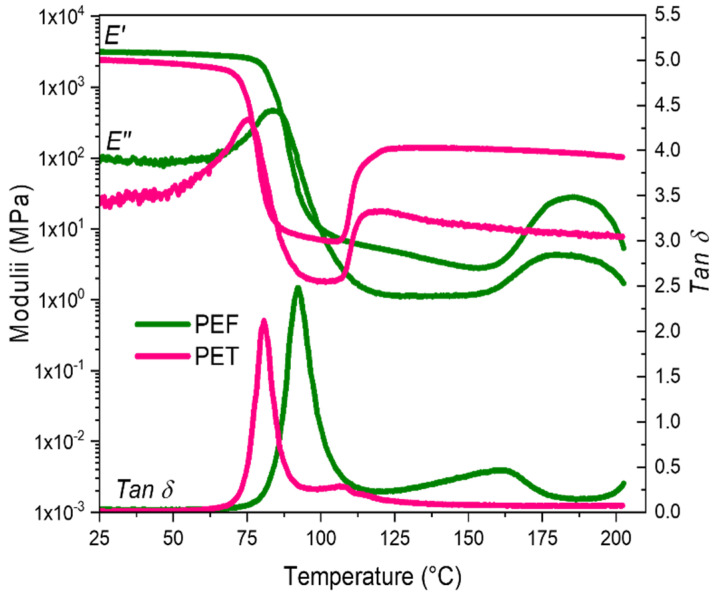
DMTA temperature scans of amorphous PEF (in green) and PET (in pink), for a heating rate of 1 °C/min and a frequency of 1 Hz, in a tensile mode [11].

To fit with the industrial protocol, the stretching has to be performed at intermediate temperatures. It means above the α-relaxation temperature, to allow chains mobility, but below the static crystallization occurrence, to only enforce strain induced crystallization. As for PET, PEF static crystallization induces a loss of formability and of transparency of the material, which is not in assessment with the industrial requirements. The forming range corresponds to the rubbery plateau of the materials. To illustrate the available forming range, a temperature scan in DMTA, from 25 °C to 210 °C at 1 Hz and 1 °C/min, is performed for PEF and PET (Figure 1).

A rapid glance to Figure 1 shows that PEF and PET amorphous rubbery plateau, which are the targeted processing ranges, do not overlap. Consequently, stretching the materials with the same set of conditions (temperature, strain rate) will not permit the efficient comparison of the stretch abilities of the two polymers nor to promote simultaneously SIC. In PEF, the low mobility of the chains and the complex interactions induced by the presence of the furan ring led to a higher α-relaxation temperature (*T_α_*), compared to PET. The α-relaxation temperatures (Figure 1), taken at the maximum of the *Tan* δ peak, are, respectively, of 80 °C for PET and of 92 °C for PEF. In parallel, the peak of the cold crystallization is detected for a temperature close to 110 °C for PET, and to 160 °C for PEF [10,11,12]. 

It appears that the cold crystallization in PEF has more difficulty in occurring than the one in PET. It has to be related with the less stability of the former, as demonstrated by the occurrence of the melting right after the static crystallization, at a significant lower melting temperature compared to PET.

Two major differences between PEF and PET must be considered for the stretching:

- PET forming range appears at lower temperature than PEF one;
- PEF exhibits a wider rubbery plateau compared to PET, and consequently a wider forming range.

It means that to perform efficient stretching in PEF, the same settings as those used in PET cannot be relevant.

#### 2.4.2. Thermal Behaviour in the Forming Range

**Figure 2:**
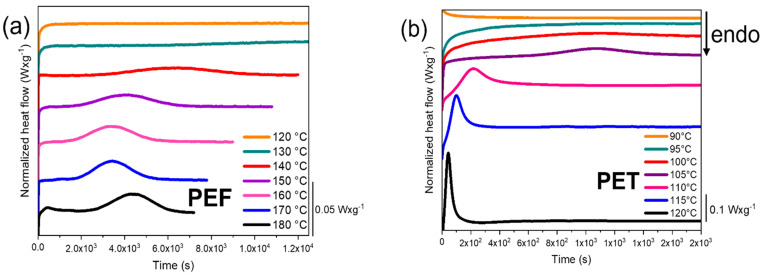
Heating scans, performed at 20 °C/min, of the previous isothermally crystallized samples for (**a**) PEF, from 25 °C to 250 °C and (**b**) PET, from 25 °C from 275 °C, measured by DSC.

**Figure 3:**
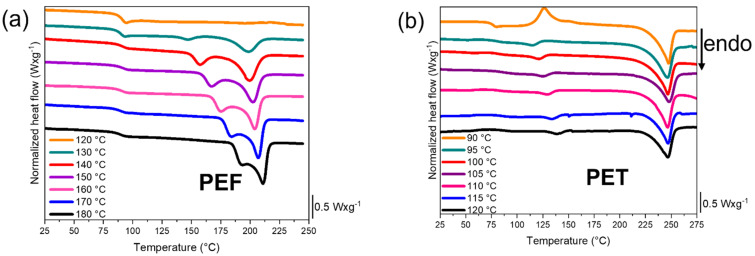
Heating scans, performed at 20 °C/min, of the previous isothermally crystallized samples for (**a**) PEF, from 25 °C to 250 °C and (**b**) PET, from 25 °C from 275 °C, measured by DSC.

Before the stretching, the samples are pre-heated for 5 min. Then, the stretching can be performed at slow or rapid strain rates. It is necessary to be sure that the materials will remain amorphous during the entire test. Thus, an estimate of the time to crystallize as a function of the temperature is needed. It allows to discriminate the sets temperature/maximum duration of test that are relevant or not. To assess heating times, the time needed to induce isothermal crystallization at temperatures included in the forming range has been measured by DSC, and is reported in Figure 2a,b, for, respectively, PEF and PET. To confirm the results of the isotherms, the heating scan of the samples submitted to the isotherms is presented in Figure 3a,b, for, respectively, PEF and PET. This heating step allows to estimate whether crystallization has been developed (through the reduction of the cold crystallization enthalpy) or initiated (through the decreasing of the cold crystallization temperature). The measurement error on the crystal ratios is estimated at ±5%. Table 1 reports the evolution of the cold crystallization, melting enthalpies and crystallinity ratios for PEF and PET. As in PEF the isotherms and the following melting of the tests performed at 120 °C and 130 °C are spread, these values are not reported. For the same reason, the values of the tests performed at 90 °C and 95 °C for PET are not reported.

Concerning PEF, with this experimental protocol, there is no evidence of crystallisation below 130 °C during the isothermal steps over 12,000 s (3 h). At 130 °C, the crystallization peak is spread but visible. For higher temperatures, the static crystallization onset appears to be higher than 1500 s. It is significantly higher than the time needed for the stretching experiments. Looking at the heating traces afterward gives additional information. Up to the isotherm at 120 °C, the heating does not reveal neither cold crystallisation nor fusion. On the contrary, the melting occurs from 130 °C which means that seemingly some nucleation can develop during the isotherms. The results are in agreement with the investigation of Martino et al. that demonstrated the formation of PEF crystals when exceeding annealing time of 1500 s and 3000 s at, respectively, 130 °C and 120 °C [46]. In conclusion, the static crystallisation is negligible in PEF up to 120 °C, whatever the test durations are. From that limit, the crystallization could be firstly initiated during the heating step, and then developed during the tensile test.

PET behaves in a different way.

For the measurement realised at 105 °C, it is visible that the isothermal crystallisation is significant from 800 s. At higher temperatures, the isothermal crystallisation occurs faster. PET is still amorphous after 1800 s at 90 °C, but semi crystalline after 1800 s at 95 °C (Figure 3b). As it was already pointed out in a previous study [47], the static crystallisation of amorphous PET can be neglected up to 90 °C. From 90 °C to 105 °C, one has to be very rigorous in terms of duration of tests as crystallisation can develop during the tests.

To be complete, the crystal ratios developed after the isotherms and the following meltings are of around 27% for PEF, and 28% for PET. It enlightens the fact that the two materials are only different in terms of kinetic of crystallization. Lastly, the melting behaviours are different between PEF and PET. After the isothermal crystallization, PET exhibits one unique melting temperature, while PEF melting appears to be multiple and sensitive to the crystallisation conditions. This trend has already been reported [5,6,45,48,49]. The stretching ranges, in terms of temperatures, are known for both materials. The strain rates must, by now, be adjusted and must fit with these temperatures.

#### 2.4.3. Dependence to the Frequency in the Forming Range

To define the stretching settings composed of couple strain rate/temperature, differences in α-relaxation temperature have to be accounted for. As the aim of this work is to compare the two polymers in an identical physical state, it has been decided to stretch the polymers with different temperatures and strain rates, but at similar equivalent strain rates at a reference temperature chosen close to the respective α-transition temperature of the materials. The WLF approach, which is the most common formal approach for time temperature principle, is used. As a result, master curves at a reference temperature are built up for PEF and PET. This approach has already been reported relevant for PEF [10,11,12,30], PET [40,50] or other materials [41,42,43,44].

From a physical aspect, this principle stipulates that the behaviour of the polymer depends in an equivalent manner on the temperature and on the strain rate. It means that starting from a given temperature, *T*, at a given strain rate, *έ* (or frequency, *f*), the mechanical behaviour will change in equivalent manners whether *T* is increased or *έ* is decreased. In a range of temperature close to *T_α_*, it is often validated that elastic modulus, *E*, is such as demonstrated in Equation (2).

(2) $$E\left(T,\;έ\right)=E\left(T_{ref},\;έ\times a_{T/T_{ref}}\right)\;\;$$

The so-called shift factor, *a_T/Tref_*, only depends on *T* and *T*_ref_, which is the so-called reference temperature. This latter can be arbitrary chosen. WLF formalism for shift factor is reminded in Equation (3) [51].

(3) $$\log\left(a_{T/Tref}\right)=\frac{-C_{1}^{0}\left(T-T_{ref}\right)}{C_{2}^{0}+\left(T-T_{ref}\right)}$$

with *a_T/Tref_* the shift factor, $C_{1}^{0}$ and $C_{2}^{0}$ (°C) the viscoelastic coefficients, *T* the temperature and *T_ref_* the reference temperature (in this work 100 °C and 90 °C for, respectively, PEF and PET).

**Figure 4:**
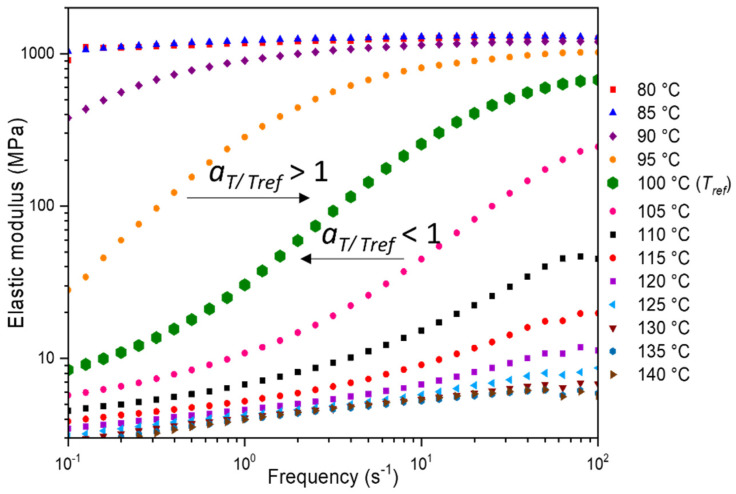
PEF frequency scans in the forming range from 10^−1^ s^−1^ to 10^2^ s^−1^, *a_T/Tref_* is the shift factor.

The master curves are deduced from isothermal frequency scans, from 1 Hz to 100 Hz. The temperature step is of 5 °C, from 85 °C to 135 °C. Only a horizontal shift is applied. These frequency scans, performed in the forming range, are visible for PEF in Figure 4.

**Figure 5:**
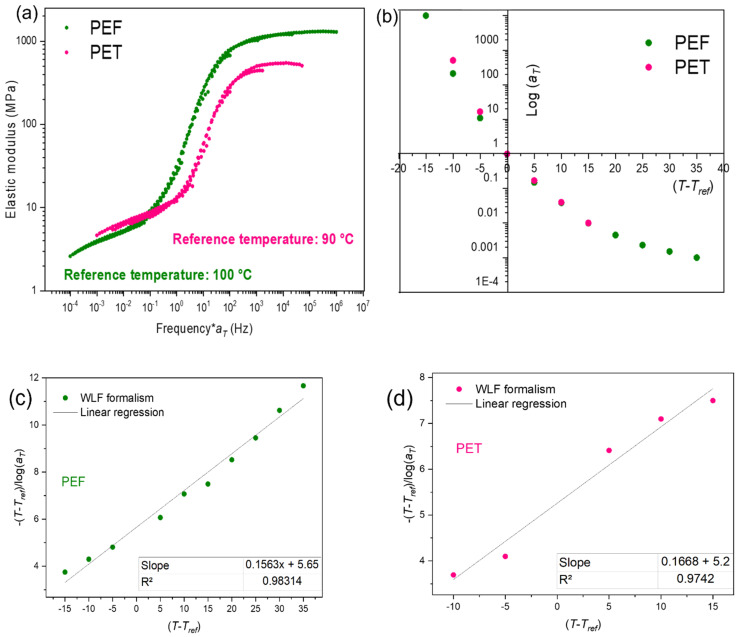
(**a**) Master curves of PEF and PET at reference temperatures of, respectively, 100 °C and 90 °C, (**b**) evolution of the shift factor as a function of the gap from the reference temperature, linear regressions of (**c**) PEF and (**d**) PET master curves used for uniaxial stretching.

According to the protocol explained, the typical PEF and PET master curves are given in Figure 5a,b depicts the evolution of the shift factor for both materials depending on the gap from the reference temperature. Figure 5c,d depict the linear regression that validates the WLF formalism of the PEF and PET master curves. From the equation of the linear regression the WLF factors ($C_{1}^{0}$ and $C_{2}^{0}$) are obtained. Table 2 summarizes the values of the WLF parameters found.

As previously settled [10,11,12], PEF and PET master curves are close. It is particularly true for their rubbery plateaux. One can observe the closeness of the *a_T_* values, between PEF and PET, once they are expressed as a function of *T − Tref* (Figure 5b). The closeness of WLF’s parameters is also visible in Table 2. It encourages thinking that the materials could be tested in the same physical state.

### 2.5. Mechanical Tests

#### 2.5.1. Exploration of the Forming Range

**Figure 6:**
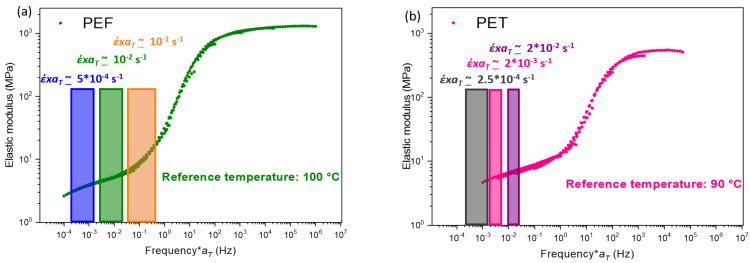
Master curves of (**a**) PEF and (**b**) PET with the areas associated to each equivalent strain rate at the reference temperature.

Once the master curves have been built, the stretching parameters can be chosen. The first step is to select the localization targeted on the master curve, represented by the equivalent strain rate at the reference temperature (*έ* x*a_T/Tref_*). It means the physical state of the material and thus, the gap from the α-relaxation. By this way, as this work aims at exploring widely the forming range of PEF and PET, three localizations have been selected on the master curve: beginning of the rubbery plateau (close to the α-relaxation), middle and end of the rubbery plateau. These zones are represented in Figure 6a,b for, respectively, PEF and PET. As a first approach, in a similar way as the Cox Merz rule [52], it is postulated that if the complex modulus evolves as a function of the frequency, the constitutive parameters of the material should evolve as a function of the strain rate [41].

For each equivalent strain rate, two different technological couples (strain rate/temperature) have been chosen. One condition is named “slow” which has to do with the slowest strain rate used, while the other condition is named “rapid” for the fastest strain rate. The aim is to have around one decade of difference on the strain rate between these two tests performed at the same equivalent strain rate. Hence, the time/temperature superposition principle is going to be tested in the large deformation domain.

The stretching settings applied during the tensile tests are, for each equivalent strain rate, summarized in Table 3.

#### 2.5.2. Stretching Device and Sample Geometry

As explained in previous works [10,11,12], a homemade device designed for film stretching under controlled temperature conditions was used. It can reproduce industrial uniaxial and biaxial stretching conditions. It is composed of four independent motor-driven arms, each coupled to a displacement sensor and a 500 N force transducer. Tensile velocity was ruled to keep strain rate, *ἐ_0_*, as constant as possible in the central zone of the samples. To this purpose, velocity of the arms varies exponentially with time.

**Figure 7:**
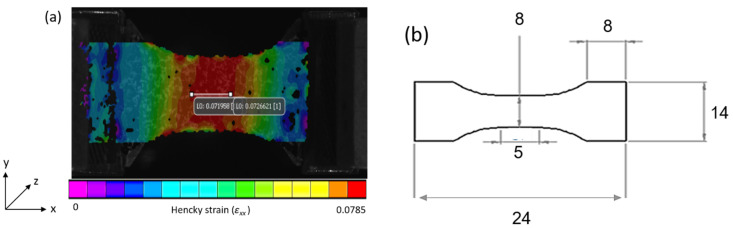
(**a**) Measurement of Hencky strain field, *ε_xx_*, during a tensile test performed at 101 °C and 0.035 s^−1^ for PEF, (**b**) Dimensions of the sample (in mm).

The sample can be heated and, after being stretched, annealed or quenched with several mobile ovens. A window of zinc selenide (ZnSe), which is partially transparent to infrared radiations, allows to measure the specimen surface temperature during the tests. On the other side of the sample, another borosilicate glass window allows local measurements of displacement fields using DIC (2D Digital Image Correlation) on painted speckle. Strain fields are computed from the displacement fields. It was shown that adding a painted speckle of thickness of almost 40 µm did not impact force measurement. DIC was used to address local Hencky’s strain *ε_xx_* and *ε_yy_*, on the specimen surface and in the two directions, longitudinal and transversal, respectively. All measurements were performed at the same location on the sample, i.e., the central zone where local stress and strain were measured. Figure 7a shows this localization, as well as an example of strain field obtained with DIC2D. Figure 7b represents the geometry of the samples.

Mechanical tests were analysed in terms of true stress (calculated using the actual instantaneous section) and true strain as depicted by Equation (4). Transverse isotropy hypothesis was assumed [10,11].

(4) $$\sigma \left(t\right)=\frac{F\left(t\right)}{e_{0}\times w_{0}\times exp\left(2\varepsilon_{yy}\left(t\right)\right)}\;$$

with, *w_0_* and *e_0_* the initial width and thickness, and *F(t)* the force evolution with time.

For each measurement, an IR pyrometer and a CCD camera were synchronised to the other analogic signals (force, displacement…). The paint was mechanically removed for post-stretching analysis. In any case, material was quenched after drawing to freeze the microstructure.

## 3. Results

### 3.1. Mechanical Behaviour

**Figure 8:**
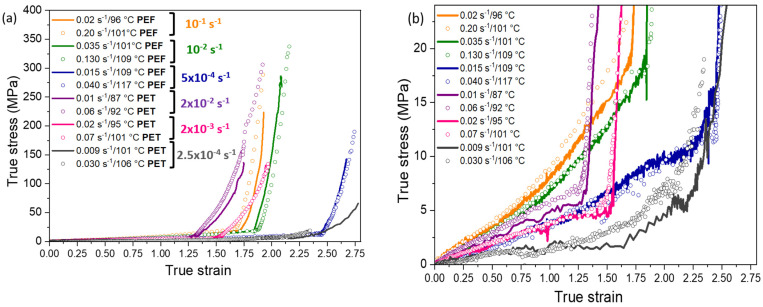
(**a**) True stress/strain curves of uniaxially stretched PEF and PET. Each colour is associated to an equivalent strain rate. Within an equivalent strain rate, lines are for “slow” tests while dots belong to “rapid” tests; (**b**) a zoom is made on the first deformation stages.

**Figure 9:**
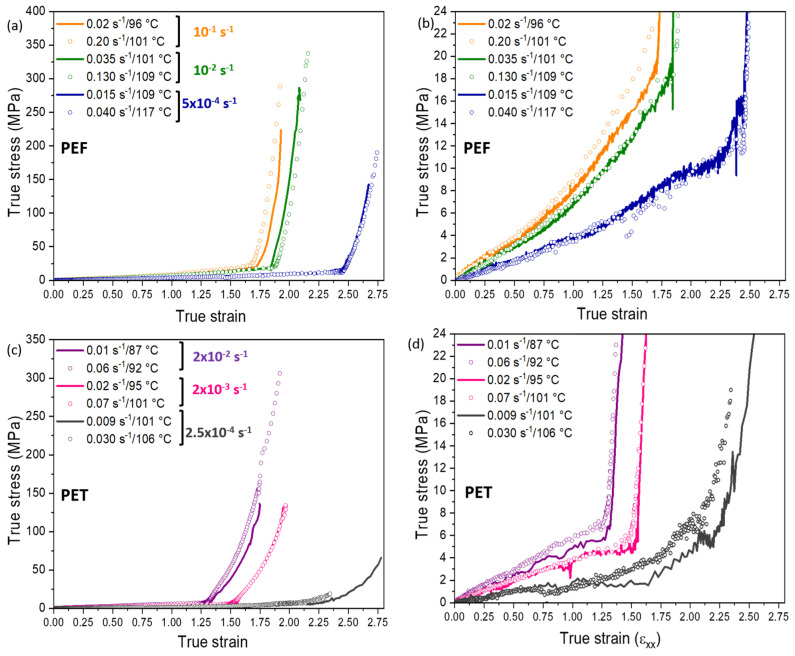
True stress/strain curves for (**a**) and (**b**) PEF, (**c**) and (**d**) PET.

Figure 8a represents the mechanical responses obtained for both PEF and PET. For a better reading of the initial steps of the stretching, a zoom is shown in Figure 8b. For the same reason, curves are redrawn in Figure 9, material by material.

The mechanical response is governed by the equivalent strain rate. The lower the equivalent strain rate, the lower the slope at the curve origin. The first stages of the stretching belong to the elastic domain. Then, the slope at the curve origin represents the Young modulus that decreases when the rubbery state of the material increases.

The stretching protocol leads to six mechanical tests for each material. Each mechanical response exhibits an impressive strain hardening up to the rupture (Figure 8). The progressive increase of the stress is due to the extension of the chains. For each equivalent strain rate tested, the stress-strain curves associated to two different couples (strain rate/temperature) are superimposed. The time/temperature principle is validated for all the conditions. It allows a potential transposition of the results to the industry that uses faster strain rates, and then higher temperatures. For the PET samples stretched with an equivalent strain rate equal to 2.5 × 10^−4^ s^−1^ (grey curves), some differences exist concerning the strain hardening onset between the two tests. For the stretching performed at 106 °C (highest temperature), it is possible that some nucleation has occurred during the pre-heating step and the test itself. In total, this sample has been heated above its α-relaxation during 335 s. Nevertheless, the isothermal tests performed in DSC close to this temperature (shown in Section 2.3) have reported no crystallization. However, the strain hardening development is complex and singular in comparison with the other tests. According to the mechanical behaviour of this sample (early apparition of the NDR, and low strain hardening level), the possible nucleation during the pre-heating step should be considered.

As shown in Figure 8b, the behaviour of PEF and PET appears really close during the first steps of the tests (except for PET stretched with an equivalent strain rate of 2.5 × 10^−4^ s^−1^). Up to around a strain of 1.3, PEF test performed at the beginning of the rubbery plateau (10^−1^ s^−1^, orange curves) exhibits the stiffer behaviour. The less rigid tests are those of PET which are localized right before the static crystallization (2.5 × 10^−4^ s^−1^, grey curves). Between them, the other tests are close.

A superimposition is even noticeable between PEF stretched at an equivalent strain rate of 5 × 10^−4^ s^−1^ (blue curves), and PET stretched at an equivalent strain rate of 2 × 10^−3^ s^−1^ (pink curves). These observations confirm that it is the gap from the α-relaxation that determines the mechanical behaviour. Indeed, to acquire the same localization on the PEF and PET rubbery plateau, and to obtain a similar response during the first stages of the stretching, around one decade of difference has to be applied on the equivalent strain rates defined at the reference temperature close to *T_α_*. It explains the superimposition of the tests performed at equivalent strain rates of, respectively, 5 × 10^−4^ s^−1^ and 2 × 10^−3^ s^−1^ for, respectively, PEF and PET.

Table 4 gathers the NDRs of each experiment, as well as the Hencky’s strain at the NDR, for PEF and PET. As the NDR apparition is relatively abrupted, the value has been determined directly at the break in slope. The reaching of high draw ratios is definitely visible.

The NDR apparition is dependent on the equivalent strain rate (and on the chain mobility). Its occurrence takes place at higher strains when the rubbery state of the material is more marked. Moreover, the NDR appears always at higher strains for PEF compared to PET, even for close localizations on their rubbery plateau. Furthermore, the strain hardening development seems sharper in PEF compared to PET. According to one of our previous works [11], the need of PEF to reach higher NDR can be due to its need to form firstly a crystal before the strain hardening occurrence. Thus, PEF is stretched and the chains have a low mobility, no intermediary phases can exist and the crystal appears only when the conditions of appearance are satisfied: (i) change of the ethylene glycols from *gauche* to *trans* (ii) the conformation of furans from *anti* to *syn*, (iii) need of two repeating units in its crystal [12,29,53])

The results obtained are in good agreement with the previous works concerning PET [11,21,40,50]. It confirms the interest of using a master curve to estimate the physical state of the material before its stretching, and then to apply the adequate couple strain rate/temperature. With the few tests chosen from the master curve, the mechanical behaviour of PEF and PET can be widely described.

The definition of the PEF stretching settings from the master curve analysis is more efficient than the choices existing in the literature [27,28,29]. In these previous works, PEF was not able to reach high level of deformations, nor to develop high level of strain hardening (the stress levels were lower than 10 MPa), and then, the induced microstructure was not a well-defined one. It is probably due to the use of a too low temperature and strain rate.

To conclude this part, the use of the protocol described in the present paper leads to control tests. It is observed that PEF and PET stretched with the relevant settings are not so different in terms of mechanical behaviours.

### 3.2. Induced Microstructure

**Figure 10:**
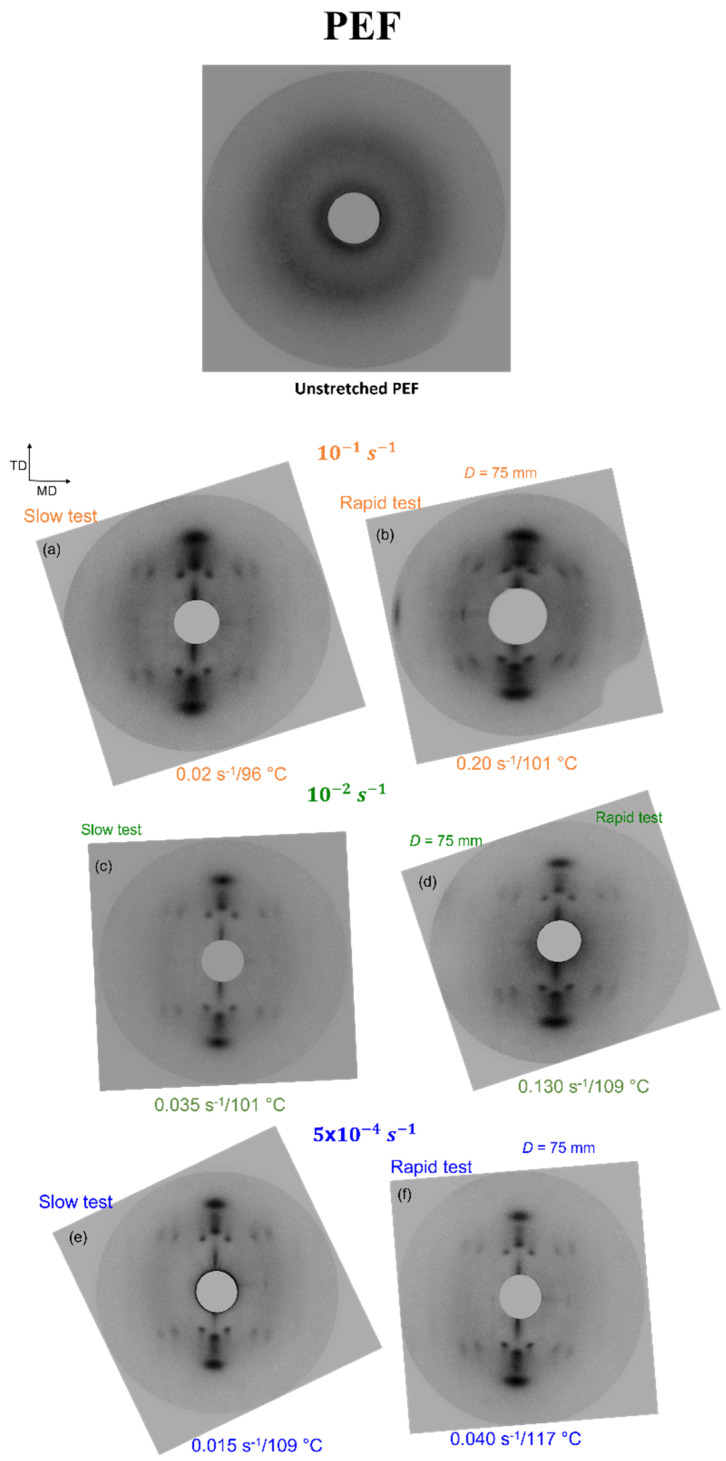
Debye-Scherrer patterns of stretched PEF, with (**a**,**c**,**e**) “slow” and (**b**,**d**,**f**) “rapid” strain rates.

**Figure 11:**
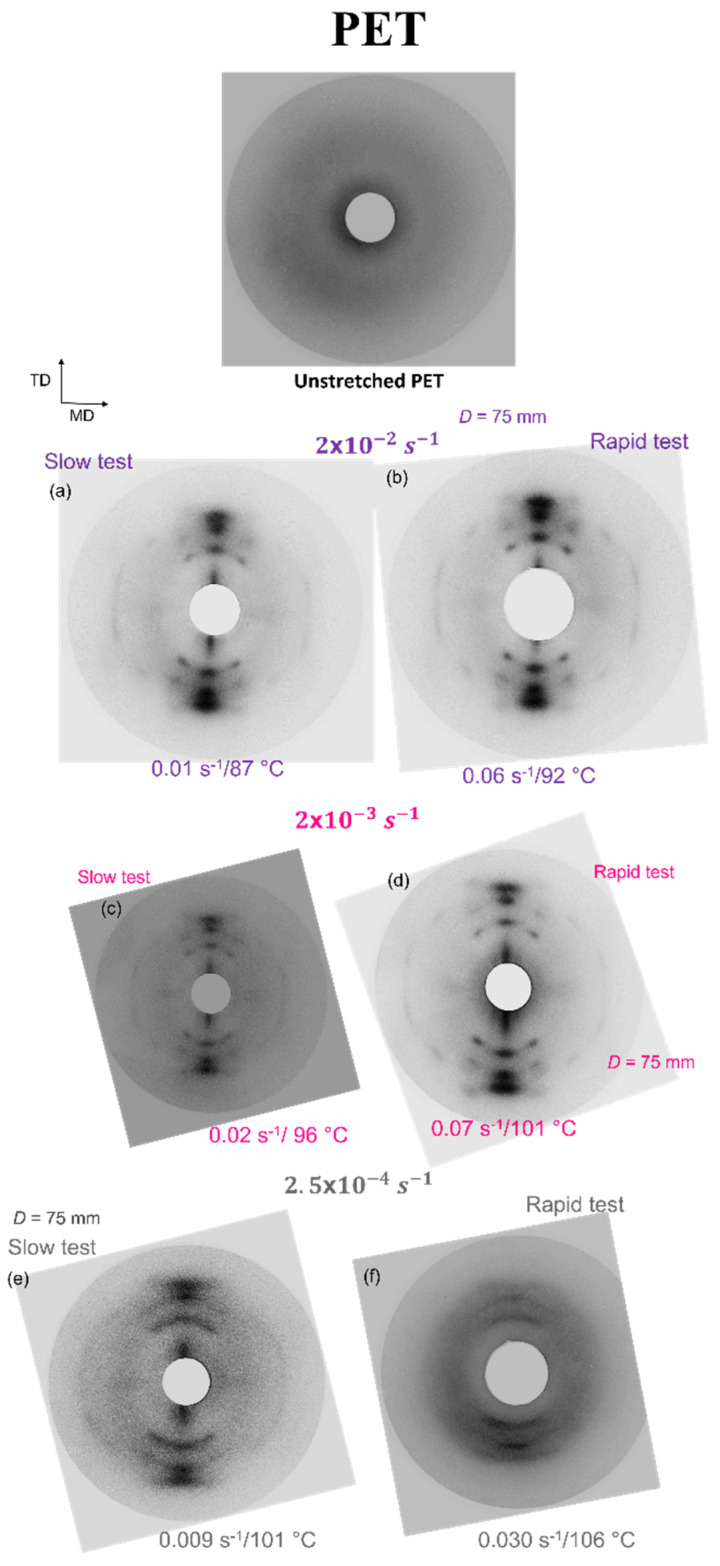
Debye-Scherrer patterns of stretched PET, with (**a**,**c**,**e**) “slow” and (**b**,**d**,**f**) “rapid” strain rates.

The creation of SIC is observable on the Debye-Scherrer pictures for PEF and PET (respectively, Figure 10 and Figure 11). Initially, the materials are amorphous and after the stretching, the observation of intense spots is obvious on the patterns. The spots represent the diffraction of the families of planes. It reveals the periodicity of the structure, oriented in the material, and thus the presence of SIC [10,11,12].

A well-defined crystal exists for all the stretching conditions. It is especially true for PEF and it validates the stretching protocol efficiency as well as the control of the stretching settings. For PET, two samples (Figure 11e,f) exhibit different patterns in comparison with the other conditions presented. The spots appear spreader and rings are partially visible. The sample stretched at 106 °C confirms this as arcs rather than spots are visible. Thus, the crystal perfection of these samples can be lower compared to the other tests. PET sample that has been stretched with the highest temperature (106 °C) can have developed a microstructure which is a mix between SIC and some nucleation occurring during the pre-heating step. It is confirmed by the low strain hardening visible in Figure 8, the microstructure of this sample is rather an organized mesophase than a crystal.

## 4. Conclusions

The aim of this work was to stretch PEF and PET all along their forming range. An original protocol was used to stretch both materials while considering their own chain mobility and thus the gap existing from the *α*-relaxation. The gap from the α-relaxation, represented through the equivalent strain rate, is the key parameter that controls the mechanical response and the NDR value.

PEF and PET can be stretched efficiently along their forming range and an important strain hardening has been noticed. SIC has been developed for all the samples. The time/temperature principle has been validated for all the stretching conditions which then allows the transposition of these results in industrial conditions. Moreover, during the first steps of the stretching, all the curves are close, and almost superimposed. It attests of the high similarity existing between PEF and PET. To acquire the same mechanical response, around one decade of difference must exist when the stretching settings are extracted from the master curve reading.

## Figures and Tables

**Table 1:** Evolution of the cold crystallization (Δ*H_c_*) and melting (Δ*H_m_*) enthalpies and crystallinity ratios (*χ*), during the isothermal treatment and the following melting of PEF and PET.

**PEF**							
*T_isotherm_* (°C)	120	130	140	150	160	170	180
Δ*H_c_* (J · g^−1^)			27.69	30.24	32.38	32.09	35.57
Δ*H_m_* (J · g^−1^)			36.53	37.86	38.22	38.79	36.92
*χ* (%)			26.09	27.04	27.30	27.70	25.85
**PET**							
*T_isotherm_* (°C)	90	95	100	105	110	115	120
Δ*H_c_* (J · g^−1^)			7.16	12.77	9.59	9.53	12.41
Δ*H_m_* (J · g^−1^)			40.13	40.58	41.08	40.65	40.53
*χ* (%)			28.66	29.00	29.34	29.03	28.95

**Table 2:** Summary of the reference temperatures, and the viscoelastic parameters of the WLF formalism for PEF and PET.

Materials	*T_ref_* (°C)	$C_{1}^{0}$	$C_{2}^{0}\;({}^{\circ}C)$
PEF	100	6.39	36.18
PET	90	5.99	31.52

**Table 3:** Summary of the stretching settings associated to each equivalent strain rate at the reference temperature, for PEF and PET.

Equivalent Strain Rate (s^−1^)	Stretching Settings		Stretching Settings	
	PEF		PET	
	Slow (s^−1^/°C)	Rapid (s^−1^/°C)	Rapid (s^−1^/°C)	Rapid (s^−1^/°C)
10^−1^	0.02/96	0.20/101		
2 × 10^−2^			0.01/87	0.06/92
10^−2^	0.035/101	0.130/109		
2 × 10^−3^			0.02/95	0.07/101
5 × 10^−4^	0.015/109	0.040/117		
2.5 × 10^−4^			0.009/101	0.030/106

**Table 4:** NDR evolution with the equivalent strain rates at the reference temperature, for PEF and PET.

**PEF**						
***έ*** × ***a_T_***	**10^−1^** × **s^−1^**		**10^−2^** × **s^−1^**		**5** × **10^−4^** × **s^−1^**	
Settings (s^−1^/°C)	0.02/96	0.20/101	0.035/101	0.130/109	0.015/109	0.040/117
Hencky’s strain at NDR	1.69	1.68	1.83	1.87	2.39	2.40
NDR (***λ***)	5.41	5.36	6.29	6.48	11.02	11.13
**PET**						
***έ* × *a_T_***	**2 × 10^−2^ × s^−1^**		**2 × 10^−3^ × s^−1^**		**2.5 × 10^−4^ × s^−1^**	
Settings (s^−1^/°C)	0.01/87	0.06/92	0.02/95	0.07/101	0.009/101	0.030/106
Hencky’s strain at NDR	1.27	1.25	1.53	1.47	2.19	2.05
NDR (***λ***)	3.56	3.49	4.61	4.34	8.93	7.76
